# Drug procurement, the Global Fund and misguided competition policies

**DOI:** 10.1186/1475-2875-8-305

**Published:** 2009-12-22

**Authors:** Richard Tren, Kimberly Hess, Roger Bate

**Affiliations:** 1 Africa Fighting Malaria, Washington, DC, USA; 2 American Enterprise Institute, Washington, DC, USA

## Abstract

In an effort to increase competition and decrease price, the Global Fund to Fight AIDS, Tuberculosis and Malaria recently began asking some grant recipients to use international competitive bidding processes for certain drug purchases. Unfortunately, for countries like Kenya, this request has caused more harm than good. After awarding the tender for its annual supply of the anti-malarial artemether-lumefantrine to the lowest bidder, Ajanta Pharma, Kenya experienced wide stock-outs in part due to the company's inability to supply the order in full and on time. Similar problems could arise in Uganda. Despite Kenya's experience, Uganda has awarded its next tender for artemether-lumefantrine to Ajanta Pharma. Uganda is already facing wide stock-outs and risks exacerbating an already dire situation the longer it takes to fulfil the procurement contract. A tender process based primarily on price cannot account for a company's ability to consistently supply sufficient product in time.

## Background

The Global Fund to Fight AIDS, Tuberculosis and Malaria (GFATM) spends millions of dollars every year to procure medicines for patients in developing countries [1]. Yet GFATM procurement policies that encourage grant recipients to procure products based largely on price alone has placed less value on quality and reliability of supply. This has led to stock-outs and questionable tenders that may cost malaria control programmes more than is saved through open tenders.

In short, GFATM is at times placing minor price reductions of a medicine above the value of health.

In countries like Kenya, where as many as 13 million people contract malaria every year and an estimated 48,000 die [2], the successful procurement and distribution of anti-malarial drugs is a matter of life and death. Given the perennial malaria risk in Kenya, drugs should always be available in all clinics. GFATM has allocated millions of dollars for the procurement of life-saving medicines, and by its own measures has done a reasonably good job ensuring that the drugs reach the right people. But its recent five-year internal evaluation acknowledges insufficient accountability of how developing countries use this money, leading to inefficient, sometimes wasteful spending [3]. In addition, inconsistent reporting by grant recipients results in unreliable data, which compounds the problem [4].

## Discussion

### The Grant-Giver

GFATM is a "financing" rather than an "implementing" body. It does not directly procure drugs, instead it invites "country coordinating mechanisms" (CCM), which may include "broad representation from governments, NGOs, civil society, multilateral and bilateral agencies and the private sector," to submit grant proposals [5].

Once approved, money is disbursed in tranches to the CCM-designated "principal recipient" (PR), who then channels money to sub-recipients. A Ministry of Health or other body may negotiate directly with a pharmaceutical company or contract with logistics firms who offer expertise in procurement. GFATM hires "local fund agents" (LFAs), usually one per grant-receiving country, who are responsible for overseeing, verifying and reporting on grant performance.

GFATM encourages its PRs to purchase drugs "in a manner that achieves the lowest possible price for products of assured quality" through competitive purchasing from qualified manufacturers and suppliers [6]. GFATM allows the procurement of drugs not yet approved by the World Health Organization (WHO) or a stringent regulatory authority (SRA) where there are fewer than two drugs available that meet one or both of these standards; these drugs should only be procured under exceptional circumstances since they have not yet passed the highest quality assessments and are possibly of inferior quality.

In the case of anti-malarial drugs, Novartis AG was the first company to produce a WHO-prequalified and SRA-approved artemisinin-based combination therapy (ACT); called Coartem^®^, it is a fixed-dose combination of artemether-lumefantrine (ALU). Today, there are eight forms of ACT approved by an SRA or the WHO, including Ajanta Pharma's ALU - Artefan^®^. For several years, GFATM's PRs contracted directly with the supplier. To increase competition, in 2007, GFATM asked some grant recipients to use international competitive bidding processes for certain drug purchases [7].

### The Kenya case

GFATM required that Kenya purchase seventy-five percent of its annual order of ALU, the recommended first-line treatment for uncomplicated malaria in Kenya, through an international open tender [7]. In line with GFATM policies, in May 2008, the Kenya Medical Supplies Agency--a GFATM sub-recipient--awarded a $12.3 million tender to Ajanta Pharma, the lowest bidder, whose ALU was, at the time, registered by GFATM as a Ci (submitted to WHO or an SRA for approval, but not yet approved). Tender documents were not made available to the authors; however, industry and academic sources indicate that the difference between Ajanta's tendered price and rival tenders was substantial, at around forty percent. The substantial cost savings from the lower bid could mitigate the risk of awarding the tender to a relatively unknown company. On the other hand, procurement agents should have questioned how realistic the pricing was and whether or not the company would be able to deliver at such a low cost. The contract stipulated that Ajanta would supply 13 million doses of Artefan in three phases to begin in October 2008 [7] at the latest, although it was widely expected that delivery would begin earlier [8].

By mid 2008, Kenya was experiencing wide stock-outs of ALU and had to place emergency orders to the President's Malaria Initiative (PMI) [9]. While PMI was able to supply the country with nearly 1.3 million doses of Coartem over July and August, Kenya's drug shortage remained.

A delayed and confusing tendering process was partly to blame for the ongoing stock-outs, but these were exacerbated and prolonged by Ajanta Pharma's inability to fulfil its contract and supply Artefan in sufficient quantities. It is possible that Ajanta would have been able to fulfil its tender for Kenya, had the Kenyan Government not delayed procurement, since Ajanta probably redirected some of its product to fulfil other contracts in the meantime; regardless, it managed its own supply poorly. Additionally, Kenyan sources tell the authors that because the product was a Ci (and hence not WHO prequalified), batch-quality testing was undertaken, which further delayed delivery. Ajanta delivered its first consignment on December 31, 2008--at least three months late. In addition, the amount that arrived was well below the expected monthly requirement, and some of the blister packages were only partially filled [10].

In March 2009, WHO issued a Notice of Concern (NOC) against Ajanta Pharma following an inspection of the company's production plant, which "revealed several major deviations from the WHO GMP [Good Manufacturing Practices] standards [11]". WHO's NOC announced the withholding of prequalification of any new Ajanta products until the deviations were satisfactorily addressed and could also consider suspending Ajanta's products that were currently listed as prequalified. Fortunately for Ajanta, the NOC has since been withdrawn [12].

### History lessons in Uganda

Despite Kenya's experience, it seems Ajanta's logistical failures have not influenced GFATM policy in neighbouring Uganda.

In early March 2009, Uganda issued an international open tender for its annual ALU supply. Fourteen companies took part, including Novartis, the large Indian generics company Cipla, and Kenya-based Cosmos (with the lowest bid) [13].

With WHO's drug quality concerns still outstanding, Uganda's Ministry of Health announced on May 8^th ^that Ajanta was the "best evaluated bidder" [14]. Though Uganda's Health Minister, Stephen Malinga, was "aware of the Kenyan situation," he told the Observer, a Ugandan newspaper, that Ajanta had "explained" itself and the Ministry had concluded Kenya's problems were "caused by the Kenya Government itself" [15]. The Ministry intended to sign a contract within five to ten days. A Ugandan news source indicated that as of early November 2009, the tender was awarded to Ajanta Pharma and Artefan would replace Coartem [16]. As of December 2009, it was unclear whether orders had actually been placed and when Ajanta was scheduled to deliver product. The delays in awarding the tender and issuing the orders have already contributed to greater shortages of ACT in Uganda.

All this leaves lingering questions about Uganda's actions; awarding a contract not to the lowest bidder or to the one with a reliable history. Tender documents on file with the authors reveal that Ajanta's tendered price was approximately four percent below that of Novartis AG, therefore there is little apparent cost mitigation to account for the added risk of procuring from a company with a poor record of delivery and an outstanding NOC at the time the open tender was issued. Furthermore, the extent to which GFATM exercises oversight and demands greater accountability over such tenders is questionable. In its guidelines, GFATM suggests PRs are responsible for oversight along the supply chain, but does not specify if or how GFATM itself is responsible, which may have contributed to the cases presented here [17].

The U.S. Government Accountability Office reported that numerous sources have raised "concerns about the quality of grant monitoring and reporting" provided by GFATM's LFAs, particularly on "their ability to assess and verify recipients' procurement capacity and program implementation." GFATM had limited access to the information it needed to manage and oversee LFAs because it did not require "systematic assessments" of their performance [18].

## Conclusions

So, is price king? GFATM is one of the most successful mechanisms for supporting malaria control programmes and is more open and transparent than almost any other multilateral or bilateral programme, but significant procurement policy problems within GFATM must be urgently addressed. Competition among suppliers will lower prices and may raise quality, but only if minimum standards are required. However, GFATM policies that have pushed competition ahead of drug quality or consistency of supply demonstrate that this does not always happen in practice. Indeed, one could argue that as much as Uganda's current procurement approach appears misguided, it is in fact in compliance with GFATM policies.

In response to queries from the authors concerning the status of medicines on GFATM's procurement list after WHO has issued an NOC, GFATM representatives indicated that they are in 'continued dialogue' with WHO, but would not de-list a medicine as long as it remained on the WHO prequalification list. However, while a WHO NOC reports on drug quality, it does not report on the ability of a manufacturer to deliver drugs, an issue that should be of concern to GFATM.

A tender process based primarily on price cannot account for a company's ability to consistently supply sufficient product in time. GFATM and its donors should insist on an assessment of the lives lost and the socioeconomic cost of Kenya's stock-out, including the cost of the emergency procurements made by the PMI. And where possible, assess how Ajanta Pharma's failures contributed to this problem.

A possible longer-term solution could be to circumvent national tendering by PRs, and instead have donors issue tenders, manage funds and deliver drugs directly to recipient countries. Such a system, however, is not only patronizing toward disease endemic countries, reducing their level of responsibility for tendering and procurement, but could also limit these countries' long-term ability to manage sustainable disease control programs. Based on Kenya's experience, it would be preferable for donors to instead issue clear procurement guidelines, giving guidance on considerations of price and logistics. Such a system should also be accompanied by a global audit of GFATM tenders and procurement. Given the fact that global taxpayer funds are used to support such tenders, a fully transparent and public audit of drug tendering and procurement should be instituted. Furthermore, in order to ensure improved tendering, tenders and details of procurement decisions, including drug prices, should be more transparent. Currently, some companies, with good intentions, publicise their product prices prior to the issuance of tenders, which allows other companies to benefit from this information when submitting their own confidential bids. A system that is more consistent with the principles of fair competition would discourage companies from publicising prices ahead of tenders and would insist on full transparency of these tenders once awarded. Lastly, to achieve improved accountability for awarded tenders, penalties for suppliers, PRs and LFAs should be instituted.

## Competing interests

The authors declare that they have no competing interests.

## Authors' contributions

RT, KH and RB contributed equally to the drafting of the text. All authors have read and approved the final manuscript.
